# Supplementary material for: Foliar application of fullerenol and zinc oxide nanoparticles improves stress resilience in drought-sensitive Arabidopsis thaliana
Source: PLoS One. 2025 Aug 19;20(8):e0330022. doi: 10.1371/journal.pone.0330022 (PMC12364364; doi:10.1371/journal.pone.0330022)
Supplement: S1 File — Supplemental Information. Analyzed drought related genes and their sequences, Supplemental Figures (S2-S3). Supplemental Tables S2-S6. Mixed-model ANOVA outputs for different analyzed datasets. (ZIP) [file pone.0330022.s001.zip › Supplemental Information.docx]

### Supplemental Information

**Foliar application of fullerenol and zinc oxide nanoparticles improves stress resilience in drought-sensitive *Arabidopsis thaliana***

Ana Joksimović^1,2^, Danijela Arsenov^3^, Milan Borišev^3^, Aleksandar Djordjevic^2^, Milan Župunski^4,*^, Ivana Borišev^2^

^1^A Bio Tech Lab Ltd; Sremska Kamenica; Serbia.

^2^Department of Chemistry, Biochemistry and Environmental Protection, Faculty of Sciences, University of Novi Sad, Serbia

^3^Department of Biology and Ecology, Faculty of Sciences, University of Novi Sad, Serbia.

^4^Institute of Cell and Interaction Biology; Heinrich Heine University; Düsseldorf; Germany

* corresponding author e-mail: [milan.zupunski@hhu.de](mailto:milan.zupunski@hhu.de)

S1 Table. Analyzed drought related genes and their sequences

| **Gene** | **Name** | **Sequence** |
| --- | --- | --- |
| AT1G45249.1_F | AREB1/ABF2_F | AGTTACAACGAAAGCAGGCAAGG |
| AT1G45249.1_R | AREB1/ABF2_R | CCTCCTTGCAGAAGATTCCTCATC |
| AT2G38310.1_F | PYL4_F | TCAGTGTTGCTCCGCCGTTATTC |
| AT2G38310.1_R | PYL4_R | ACGGACCAAACGGTGGAGATTG |
| AT2G38340.1_F | DREB19_F | TGAGTCACCGTGGTGCAAACTC |
| AT2G38340.1_R | DREB19_R | CTGCAGTAGCAAACGTGCCAAG |
| AT3G11020.1_F | DREB2B_F | ATGAAGCGGCTACCGCTATGTACG |
| AT3G11020.1_R | DREB2B_R | TCAGACCCAACAGACTGAGGGAAG |
| AT3G11410.1_F | PP2CA_F | TCCTCTCTCCGTAGATCACAAGCC |
| AT3G11410.1_R | PP2CA_R | GGCAAGAACTCCAAGAACCCTAGC |
| AT3G18780.2_F | ACT2_F | TCTTCCGCTCTTTCTTTCCAAGC |
| AT3G18780.2_R | ACT2_R | ACCATTGTCACACACGATTGGTTG |
| AT4G17870.1_F | PYR1_F | TTACCGGCGAACACATCAACGG |
| AT4G17870.1_R | PYR1_R | ATCCGGTAACTCTCCGTTCGTC |
| AT4G34000.1_F | ABF3_F | GTTCTCAACCTGCAACACAGTGC |
| AT4G34000.1_R | ABF3_R | TCCAGGAGATACTGCTGCAACC |
| AT5G05410.1_F | DREB2A_F | TGTCTGGAGAATGGTGCGGAAG |
| AT5G05410.1_R | DREB2A_R | TCGCTCAGCCAATGCTTATCCG |
| AT5G52300.1_F | RD29B_F | ACTGATCCCACGCATAAAGGTG |
| AT5G52300.1_R | RD29B_R | CTCGTCGGAAAGTCTTCTTCGC |
| AT5G57050.1_F | ABI2_F | CTCGCAATGTCAAGATCCATTGGC |
| AT5G57050.1_R | ABI2_R | TTACTCGCCGCACTGAAGTCAC |
| AT5G52310.1_F | RD29a_F | TGGACAAAGCAATGAGCATGAGC |
| AT5G52310.1_R | RD29a_R | AGGTTTACCTGTTACGCCTGGTG |

**
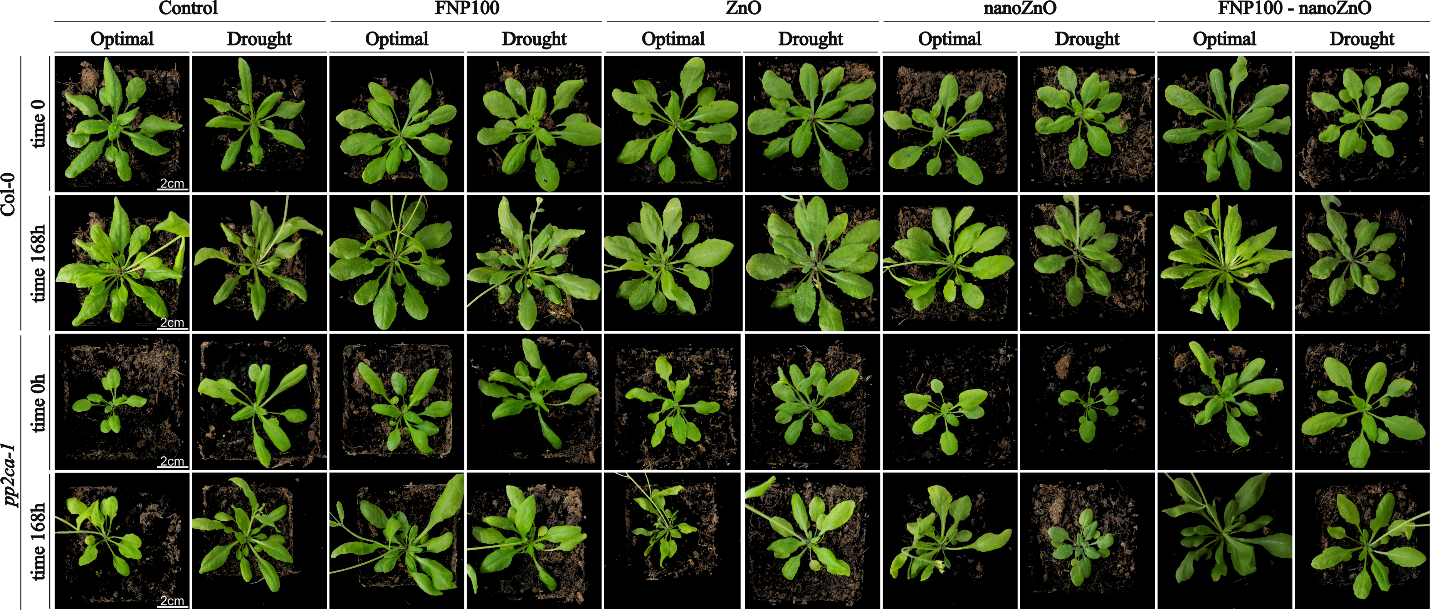
**

S1 Fig. Leaf rosette phenotypes

**
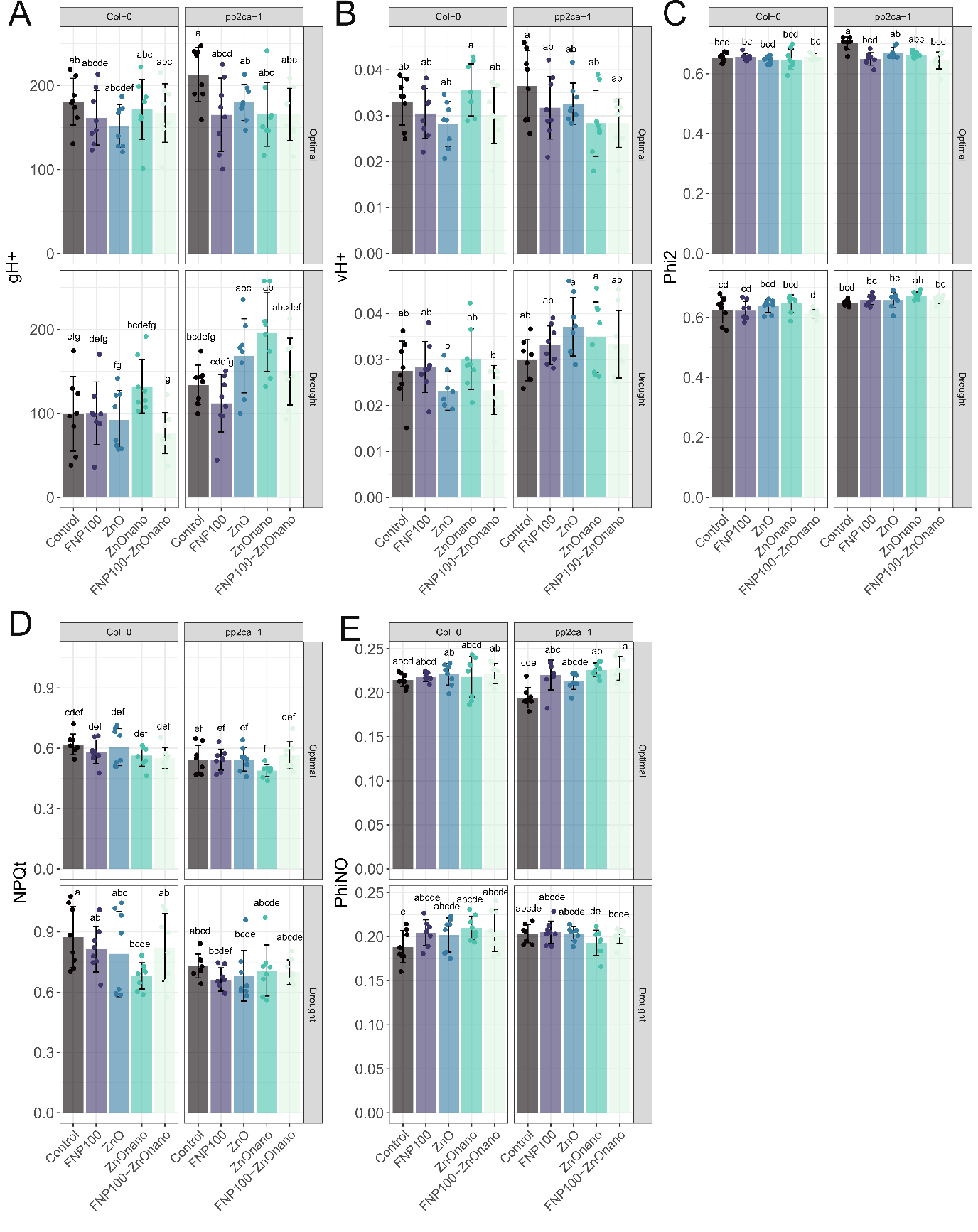
**

S2 Fig. Chlorohyll florescence parameters**.** gH+ (thylakoid membrane conductivity to protons), vH+ (steady-state proton flux), Phi2 (quantum yield of photosystem II), Fv/Fm (variable-to-maximal fluorescence ratio), PhiNQ (non-regulatory energy dissipation), and NPQt (non-photochemical quenching)

**
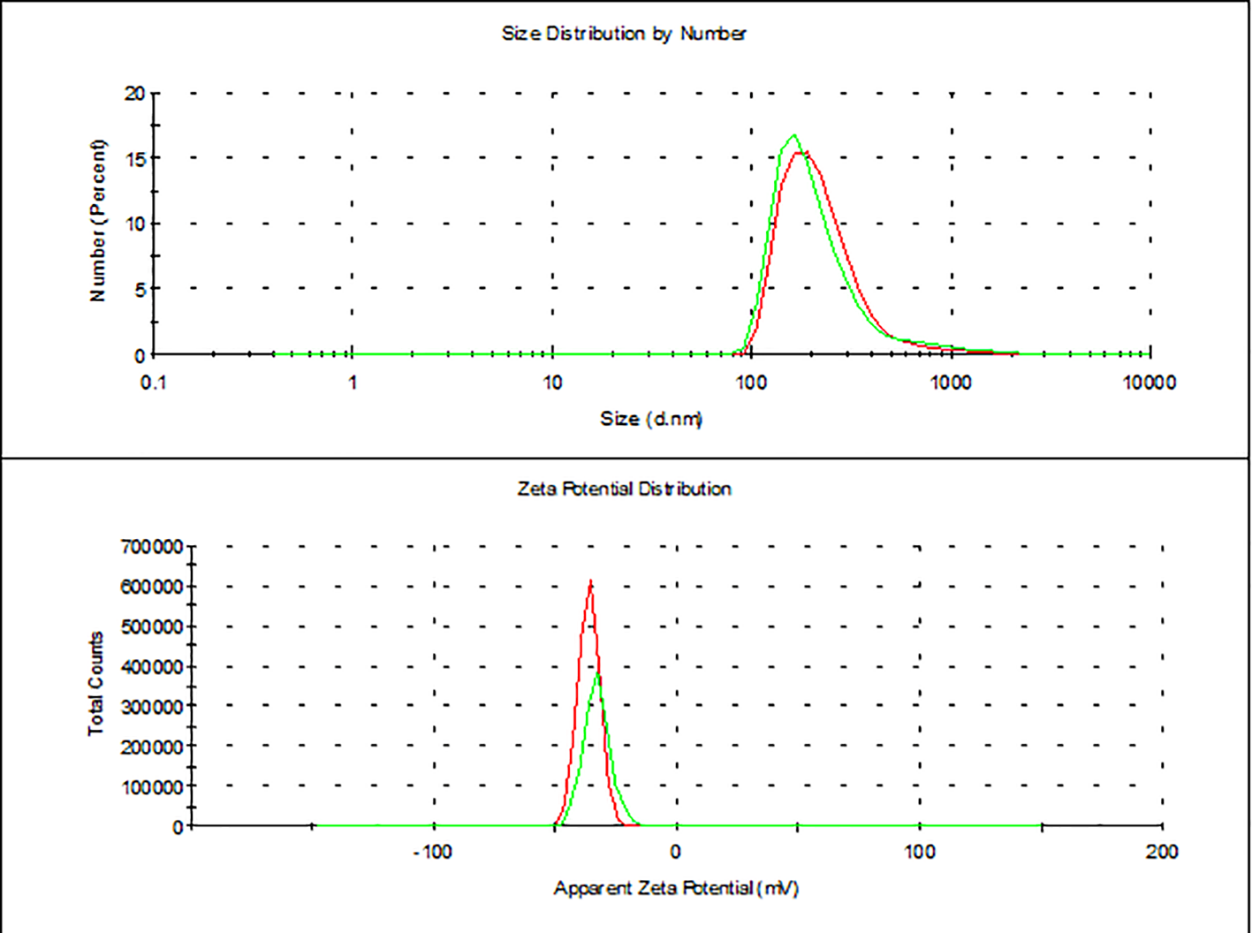
**

S3 Fig. DLS and zeta potential analyses. Particle size distributions by number:

a) FNP100-ZnOnano combination at a molar ratio of FNP 100 μmol/nano ZnO 1.3 μmol, after 24 hours, in aqueous solution (green line, ZnO as referent material and red line, FNP as referent material);

b) ζ potential of FNP100-ZnOnano combination at a molar ratio of FNP 100 μmol/nano ZnO 1.3 μmol (red line, ZnO as referent material and green line, FNP as referent material), after 24 hours, in aqueous solution.
