# Supplementary material for: Foliar application of fullerenol and zinc oxide nanoparticles improves stress resilience in drought-sensitive Arabidopsis thaliana
Source: PLoS One. 2025 Aug 19;20(8):e0330022. doi: 10.1371/journal.pone.0330022 (PMC12364364; doi:10.1371/journal.pone.0330022)
Supplement: Attachment — Submitted filename: Response to Reviewers PONE-D-25-20535.pdf [file pone.0330022.s005.pdf]

Rebuttal letter

We sincerely thank the editor and reviewers for their constructive comments and thoughtful suggestions, which have significantly contributed to the improvement of our manuscript's quality. We believe that the revised version now meets the standards and publication criteria of *PLOS ONE*. All suggested revisions have been carefully addressed, and the updated manuscript includes a marked-up version with all changes clearly highlighted.

Reviewer #1: Summary of Major Comments (Overall Paper):

Clarification and strengthening of key mechanisms: Ensure the discussion addresses specific physiological and molecular pathways more clearly, with emphasis on how the nanoparticles' physicochemical properties impact drought stress at the cellular and molecular levels.

We thank the reviewer for this valuable feedback. We have addressed this point by incorporating additional mechanistic insights into our discussion. Specifically, we have clarified the physiological and molecular pathways involved and emphasized how the physicochemical properties of the nanoparticles influence drought stress responses at both cellular and molecular levels.

"Overall, the unique physicochemical properties of ENPs, such as a small size, a large surface area, high cation exchange capacity (CEC), high absorbability, and potent free radical scavenging capacity, ultimately contribute to the plant's ability to withstand drought stress [1,4]. "

"Fullerenol-induced modulation of ABA metabolism was previously reported by Xiong et al. (2018), who demonstrated that under drought stress, the application of 1 mg L^{-1} fullerenol significantly upregulated the expression of NCED3, a key gene in ABA biosynthesis, while downregulating CYP707A3, which is involved in ABA catabolism, in *Brassica napus* [15]. These results suggest that FNP's impact on plant metabolism is partly mediated through ABA-regulatory signaling cascades."

Further exploration of synergy: Given that the combination of FNP and ZnO nano has a synergistic effect, ensure that this interaction is clearly explained in both the results and discussion sections.
We thank the reviewer for this insightful suggestion. In response, we have revised the Results

and Discussion section to explicitly highlight the interactive and synergistic impacts of the combined treatment. Specifically, we now elaborate on how the joint application leads to enhanced physiological traits (e.g., improved CO_2 assimilation, modified stress biomarkers accumulation, and antioxidant enzyme activities) and modulates gene expression in a more pronounced manner than individual treatments. We have added the following paragraph to the revised manuscript.

"Additionally, to the best of our knowledge, this is the first study to investigate the synergistic action of fullerenol and ZnO nanoparticles applied foliarly to mitigate drought stress, thereby opening a novel direction for nano-enabled plant resilience strategies. It is essential to emphasize that the FNP-ZnO nano combined treatment revealed a synergistic effect driven by the complementary mechanisms of both applied nanoparticles. While FNP exhibits strong hygroscopic and antioxidant properties, enabling the modulation of cellular water potential and enhancing ROS scavenging under stress conditions [6, 66], ZnO nanoparticles, on the other hand, may serve as sources of micronutrients and redox modulators, influencing osmotic balance, ion homeostasis, and oxidative signaling [23, 68, 69]. The superior performances were evident in boosting photosynthetic performance (Fig. 7 B, D) and minimizing oxidative damage under the combined treatment (Fig. 8B, C, E), suggesting an additive modulation of key drought-related pathways."

Experimental considerations: If there are limitations in experimental design (e.g., the use of a single model plant like Arabidopsis), these should be discussed, and potential extensions to other crops or field studies could be suggested.

We thank the reviewer for this insightful suggestion. *Arabidopsis thaliana* was chosen as a model plant based on its well-characterized genome, established stress-response pathways, and suitability for controlled mechanistic studies. Its use allowed for precise monitoring of physiological, biochemical, and molecular responses to nanoparticle treatment. Moreover, in this study, we have used ecotype Columbia (Col-0) wild type and *pp2ca-1* insertional mutant, which encodes the Protein Phosphatase 2 C(PP2C), a negative regulator of the abscisic acid signaling pathway. Since PP2Ca is a negative regulator of the abscisic acid pathway, plants typically respond more to drought stress when this gene is disrupted, as described in the Materials and Methods section.

However, we agree that validation in agriculturally relevant crops and field conditions is essential for broader applicability. We have now included a brief discussion in the revised

manuscript that emphasizes this point and suggests that future research should focus on extending these findings to economically important crops in real-world agronomic settings.

"Even though the results of this study are elucidated on *Arabidopsis thaliana*, which served as an effective model for elucidating the physiological and molecular mechanisms of nanoparticle action, further studies are needed to validate these findings in crop species under agronomically relevant conditions. Such translational research is essential for determining the practical applicability of nanoparticle-based stress mitigation strategies."

Suggestions for scalability and practical applications: Providing a more explicit connection between the findings and real-world applications, including challenges to scalability, would improve the practical relevance of the paper.

We appreciate the reviewer's insightful comment regarding the need to strengthen the connection between our findings and their real-world applicability. In response, we have revised the Discussion section to more explicitly address potential challenges and considerations related to scalability, including nanoparticle formulation stability and environmental safety under field conditions. We have added the following sentences to the revised manuscript.

"Although the beneficial effects of ZnO nanoparticles, fullerenol, and their combination have been demonstrated under controlled conditions, scaling these approaches to field-level agriculture requires addressing challenges such as application methods, formulation stability, environmental persistence, and cost-effectiveness. Additionally, future research should assess long-term ecological impacts and consider regulatory frameworks to ensure their safe and sustainable use in commercial crop production."

Acknowledgment of unconfirmed mechanisms: Future studies to better define the mechanisms and explore other potential interactions (like the role of antioxidant properties) should be clearly outlined.

We appreciate the reviewer's insightful suggestion regarding the need to acknowledge unconfirmed mechanisms more clearly. In response, we have revised the discussion section to include an explicit statement emphasizing the need for future studies to better define the molecular and physiological mechanisms of action, particularly those related to potential

nanoparticle involvement in ABA signaling network. This addition helps clarify the scope of our conclusions and highlights directions for further research.

"Moreover, considering the current limitation in understanding how nanoparticles trigger both ABA-dependent and ABA-independent stress signaling pathways, additional mechanistic studies are necessary to clarify these processes and evaluate the role of nanoparticles in other hormonal pathways."

Statistical rigor and data transparency: Ensure statistical methods and data representation are transparent and appropriately described, as this strengthens the reliability of the conclusions.

We appreciate the reviewer's emphasis on statistical rigor and data transparency. We would like to highlight that detailed information on statistical methods, including mixed-effects modeling, Type II ANOVA, post hoc comparisons, and PCA, is already provided in the "Data Analysis" section of the manuscript. Also, in the Materials and Methods section, we have included the sample size and number of replicates for each analysis. Additionally, we have ensured full data transparency by including the raw data used for all analyses as part of the Supplementary Information (Table "DATASET").

Reviewer #2

The current MS entitled " Foliar application of fullerenol and zinc oxide nanoparticles improves stress resilience in drought- sensitive Arabidopsis thaliana". In this sense, manuscript's is appropriate in order to represents the the subject of the research. The abstract of the manuscript is precise and present whole manuscript objectives. The references used in the manuscript are up- to-date ,satisfactory and convenient. The conclusion should only consist of key findings and significance.

We thank the reviewer for this helpful suggestion. In response, we have revised the Conclusion section and retained only the key findings and scientific significance. The revised version now only focuses on the main results, their implications, and the potential for future applications.

"This study demonstrates for the first time that foliar application of low-dose ZnO nanoparticles (10 mg/L), alone or in combination with fullerenol nanoparticles (FNP), enhances drought tolerance in *Arabidopsis thaliana*. The observed improvements in redox balance, photosynthetic performance, and stomatal regulation point to multiple physiological pathways to support drought acclimation. At the molecular level,

distinct gene expression patterns suggest involvement of both ABA-dependent and independent pathways. Notably, the synergistic effect of the combined application underscores the potential of nanoparticle-based biostimulants in plant stress management. These findings offer a promising direction for future research in nanotechnology-driven strategies for sustainable agriculture under climate stress."

Reviewer #3

The manuscript "Foliar application of fullerenol and zinc oxide nanoparticles improves stress resilience in drought-sensitive Arabidopsis thaliana" presents a detailed and thorough study about characterization and chemical properties of fullerenol nanoparticles (FNP) and zinc oxide nanoparticles (ZnO nano). Arabidopsis thaliana is an interesting choice to undertake these studies as it is regarded as model plant to study biology, stress response and genetics. As a result of nanoparticle treatment, study about changes in gene expression emphasized synergistic impact of combining ZnO nanoparticles with FNPs. However, a few queries need to be addressed to make this manuscript more rigorous.

1. Hydroxylated fullerenes have already been studied extensively for foliar applications previously. What leads to choice of fullerenol in your research?

We appreciate the reviewer's valuable question. Our decision to use foliary applied fullerenol in this research lies in lack of studies dealing with more mechanistic approach on investigating the effects of fullerenol in model plant species:

Among fullerenols, our FNP (fullerenol nanoparticles, synthesized in our lab) was selected due to the more accessible initial experimental framework it offers for elucidating the mechanisms of nanoparticle action, particularly considering our decades-long experience in studying the effects of FNP applications on various biological models. The key challenge of this research lies precisely in investigating the impact of our FNP on a genetically defined model plant, Arabidopsis—an approach undertaken for the first time in this study.

2. In introduction, are ENPs, engineered nanoparticles? Explain a little about ENPs to make the readers better understand them.

Yes, we apologize for this oversight. We have defined the term.

"Engineered nanoparticles (ENPs) are purpose-designed nanomaterials with at least one dimension below 100 nm, exhibiting tailored chemical and physical properties. Composed of metals, polymers, semiconductors, or carbon-based materials, ENPs are widely used in biomedicine, electronics, environmental, and agricultural applications. "

3. What is the rationale behind choosing fullerenes with 24 hydroxyl groups? As 18 to 40 hydroxyl groups is considered the practical range for most applications, why did the study not consider fullerenols with different numbers of functional groups?

We thank the reviewer for the opportunity to clarify this point. We added the following sentences to explain the issue in the additionally manuscript:

"The synthesized FNP used in this study contains 24 hydroxyl groups - a form with which we have extensive experience across various *in vitro* and *in vivo* biological models. Fullerenols bearing 18 or 40 hydroxyl groups differ in both structural organization and surface charge characteristics compared to the 24-hydroxylated FNP, which can ultimately result in distinct biological responses."

4. Under the heading of Chemicals Used, synthesis of fullerenol NPs has not been explained. It is not appropriate to present it as a reference.

We thank the reviewer for the opportunity to provide further details regarding the synthesis of the FNP used in this study, and added the following text to the manuscript:

"The fullerenol containing 24 hydroxyl groups ($C_{60}(OH)_{24}$) was synthesized through a two-step chemical process. In the first step, catalytic bromination of C_{60} with elemental bromine was carried out in the presence of $FeBr_3$. This reaction yields a single reaction product, $C_{60}Br_{24}$, without occluded bromine, and with well-defined *Th* symmetry [31]. In the second step, the bromine atoms were fully substituted by hydroxyl groups through a reaction carried out in an alkaline aqueous environment. The resulting reaction product is a molecule exhibiting high symmetry with a defined arrangement of –OH groups [8]. In aqueous solution, the fullerol molecules form stable nanoparticles with a zeta potential of approximately -25 mV to -50 mV [16, 31, 66]."

5. In topic, RNA Isolation and quantitative RT-PCR analysis, how the RNA quality and purity ensured?

We thank the reviewer for their insightful comment. As stated in the "RNA Isolation and quantitative RT-PCR analysis" subsection, we initially assessed RNA quality and purity using a NanoDrop spectrophotometer by monitoring A260/280 and A260/230 ratios. We also introduced an additional quality control step by assessing the resulting cDNA with a Qubit fluorometer. Only samples meeting acceptable purity criteria (A260/280 > 1.7 and A260/230 > 2.2) were retained for qRT-PCR analysis.

This way of quality check is a standard method in plant biology studies, not just for qPCR experiments, but for RNAseq experiments as well.

6. The results of SEM, DLS, Zeta potential, XRD Diffractograms, and FTIR of the FNP100-ZnO nano combination effectively characterize their formation.

We appreciate the reviewer's valuable comment.

We thank the reviewer for this valuable and affirmative comment. In addition to evaluating the biological effects, we aimed to monitor and confirm the chemical behavior of the nanoparticles investigated in the study. The SEM, DLS, Zeta potential, XRD, and FTIR analyses were therefore included to ensure comprehensive physicochemical characterization of the FNP100-ZnO nanoformulation.

7. During experiment were TBARS levels correlated statistically with non-enzymatic antioxidants like proline/glutathione?

We thank the reviewer for this insightful question. We did not perform statistical correlation analyses between TBARS levels and non-enzymatic antioxidants such as proline and glutathione. Our observations are based on concurrent trends observed across treatments. Accordingly, we have revised the sentence to avoid overinterpretation.

"We observed that shifts in catalase and ascorbate-peroxidase activities aligned with reductions in proline and glutathione spikes under drought stress, further supported by decreased TBARS levels, an oxidative stress indicator."

8. Effects of different ZnO nanoparticle concentrations on plant growth and stress resistance are reviewed extensively, quoting numerous references. It looks like little overemphasized in comparison to FNP100-ZnO nano combination.

We thank the reviewer for this observation. We agree that the section discussing ZnO nanoparticles includes a greater number of cited studies, reflecting the broader existing literature on ZnO nanoparticles in plant systems. In contrast, to the best of our knowledge, our study is the first to report on the combined foliar application of fullerenol and ZnO nanoparticles and their synergistic effects on drought stress mitigation in *Arabidopsis thaliana*. To address the reviewer's concern and better balance the discussion, we have revised the Discussion section to emphasize the novelty and significance of our findings related to the FNP100-ZnO nano combination, particularly in highlighting their unique synergistic impact on photosynthetic regulation, antioxidant responses, and gene expression. These revisions clarify how the combined treatment represents an innovative and promising direction for nanotechnology applications in plant stress physiology.

"Additionally, to the best of our knowledge, this is the first study to investigate the synergistic action of fullerenol and ZnO nanoparticles applied foliarly to mitigate drought stress, thereby opening a novel direction for nano-enabled plant resilience strategies. It is essential to emphasize that the FNP-ZnO nano combined treatment revealed a synergistic effect driven by the complementary mechanisms of both applied nanoparticles. While FNP exhibits strong hygroscopic and antioxidant properties, enabling the modulation of cellular water potential and enhancing ROS scavenging under stress conditions [6, 66], ZnO nanoparticles, on the other hand, may serve as sources of micronutrients and redox modulators, influencing osmotic balance, ion homeostasis, and oxidative signaling [23, 68, 69]. The superior performances were evident in boosting photosynthetic performance (Fig. 7 B, D) and minimizing oxidative damage under the combined treatment (Fig. 8B, C, E), suggesting an additive modulation of key drought-related pathways."

9. What is the rationale behind excluding any other plant hormone pathways, like auxin or salicylic acid for studying the effects of nanoparticles on plant stress responses?

We thank the reviewer for this observation. Our primary focus was on the abscisic acid (ABA) pathway because ABA is the key hormone involved in mediating plant responses to drought stress. ABA is involved in stomata closure, thus being at the forefront of fast drought induced responses. By concentrating on ABA, we aimed to elucidate the specific effects of nanoparticles on this central regulatory pathway. Additionally, we wanted to investigate

whether fullerenol and its interaction with ZnO nanoparticles influence plant stress responses through ABA-dependent or ABA-independent mechanisms. We agree with the reviewer that other hormones, such as auxin or salicylic acid, also play essential roles in stress responses; however, exploring these pathways was beyond the scope of the current study. We acknowledge that future research could expand on this by examining the interactions between nanoparticles and other hormonal pathways.

"Moreover, considering the current limitation in understanding how nanoparticles trigger both ABA-dependent and ABA-independent stress signaling pathways, additional mechanistic studies are necessary to clarify these processes and evaluate the role of nanoparticles in other hormonal pathways."

Reviewer #4

This manuscript explores the physiological, biochemical, and molecular effects of foliar application of fullerenol nanoparticles (FNP) and zinc oxide nanoparticles (ZnO NPs), alone and in combination, on drought-stressed Arabidopsis thaliana (Col-0 and pp2ca-1 mutant). The study suggests that both nanoparticles, especially FNP, enhance drought tolerance by modulating antioxidant enzyme activities, gene expression related to ABA signaling, and water use efficiency. The manuscript has strong potential but requires major revisions to address critical issues related to mechanistic interpretation and statistical clarity. I recommend a major revision and encourage the authors to significantly enhance the methodological clarity and depth of the discussion.

1. Gene expression results are not convincingly linked to phenotype. The expression data for ABA-dependent and independent pathways are descriptive but lack a direct connection to the observed physiological effects. Time-course experiments would provide a clearer mechanistic understanding of gene regulation under drought and NP treatment.

We thank the reviewer for this insightful observation. In the revised discussion section, we have elaborated more explicitly on the connection between the expression profiles of ABA-dependent and ABA-independent genes and the observed physiological outcomes, such as changes in stomatal conductance, CO_2 assimilation, and antioxidant activities. Specifically, we now highlight how the downregulation of PP2CA and ABI2 under drought correlates with

increased stomatal conductance and photosynthetic efficiency, suggesting that ABA signaling attenuation contributes to enhanced drought resilience. Similarly, we discuss how the upregulation of DREB genes aligns with reduced oxidative stress markers and elevated antioxidative enzyme activity, indicating a link between transcriptional activation and redox homeostasis. While we agree that time-course experiments would provide a more detailed mechanistic resolution, our findings nonetheless suggest functional associations between transcriptional shifts and phenotypic adaptation. We have acknowledged this limitation in the discussion and proposed it as a direction for future research.

"These molecular responses, including the downregulation of PP2CA and ABI2 under drought conditions, are reflected in physiological phenotypes such as increased stomatal conductance and improved CO_2 assimilation (Fig. 7). This suggests that modulation of ABA signaling plays a key role in enhancing drought resilience, as evidenced by the observed phenotypic outcomes."

"Furthermore, the upregulation of DREB genes in FNP-treated ABA-hypersensitive mutants under drought stress corresponds with the reduction of oxidative stress markers and an increase in antioxidative enzyme activity (Fig. 8), indicating its functional role in ROS scavenging. "

"Although it was previously stated that FNP mediates changes in both early and late responses to osmotic stress [89], time-course experiments would provide a more detailed mechanistic resolution of gene regulation under drought and FNP and ZnO-nano treatments."

2. Nanoparticles can accumulate or trigger unintended responses. No analysis is provided for potential phytotoxicity, metal accumulation, or long-term side effects in the plants. Authors must include at least a discussion of possible NP toxicity or ecological implications.

We appreciate the reviewer's concern regarding the potential phytotoxicity and ecological implications of nanoparticle use. In response, we would like to clarify that we have explicitly addressed these concerns in the discussion section, emphasizing the dual nature of nanoparticle effects depending on concentration, mode of application, and nanoparticle composition.

We have discussed previous reports demonstrating the toxicity of ZnO nanoparticles under root application and at high concentrations, referencing numerous studies by Khan et al. (2019), Lee et al. (2009), and others [83–86]. These findings were contrasted with our own use of low-concentration (10 mg/L) foliar applications, which are below toxicity thresholds

reported in Arabidopsis. We have emphasized that foliar application typically leads to lower accumulation and reduced systemic toxicity compared to soil or hydroponic exposure. Similarly, we highlighted prior work indicating that fullerenol toxicity is inversely related to hydroxylation degree, and our study used highly hydroxylated fullerenols ($C_{60}(OH)_{24}$), which are known to have low cytotoxicity and higher antioxidant activity (Kovel et al. 2021). Additionally, we have revised the manuscript by adding a brief discussion regarding long-term impact and ecological implications.

"Although the beneficial effects of ZnO nanoparticles, fullerenol, and their combination have been demonstrated under controlled conditions, scaling these approaches to field-level agriculture requires addressing challenges such as application methods, formulation stability, cost-effectiveness, environmental persistence, and potential trophic transfer risks. Additionally, future research should assess long-term ecological impacts and consider regulatory frameworks to ensure their safe and sustainable use in commercial crop production. Moreover, considering the current limitation in understanding how nanoparticles trigger both ABA-dependent and ABA-independent stress signaling pathways, additional mechanistic studies are necessary to clarify these processes and evaluate the role of nanoparticles in other hormonal pathways."

3. While the discussion is extensive, it often summarizes results without offering mechanistic insights. How exactly do FNPs enhance water retention or trigger ABA pathways?

We thank the reviewer for this valuable suggestion. In response, we have revised the *Discussion* section to include additional mechanistic insights into how FNP may influence ABA-related pathways. Specifically, we now reference previous findings by Xiong et al. (2018), which demonstrated that fullerenol treatment under drought stress upregulated *NCED3*, a key gene in ABA biosynthesis, and downregulated *CYP707A3*, which is involved in ABA catabolism in *Brassica napus*. This evidence supports the hypothesis that FNPs may modulate ABA metabolism and signaling.

"Fullerenol-induced modulation of ABA metabolism was previously reported by Xiong et al. (2018), who demonstrated that under drought stress, the application of 1 mg L^{-1} fullerenol significantly upregulated the expression of NCED3, a key gene in ABA biosynthesis, while downregulating CYP707A3, which is involved in ABA catabolism, in *Brassica napus* [15]. These results suggest that FNP's impact on plant metabolism is partly mediated through ABA-regulatory signaling cascades."

4. “FNPs act as water sponges and gradually release water under drought...” This hypothesis lacks experimental confirmation in the current study. No direct measurement of water retention or release by FNPs in planta is presented.

We agree with the reviewer that this hypothesis is not directly confirmed within the current experimental setup. Accordingly, we have clarified this in the revised manuscript by explicitly stating that this mechanism is a proposed explanation supported by our previous findings, rather than an experimentally verified outcome in the present study. The statement has been revised.

“Although not experimentally confirmed in this study, previous work by our coauthors has shown that water molecules interlock within fullerenol self-assembled aggregates, with notable water release from solvation layers occurring when temperatures rise above 36°C [66].“

5. “RD29A exhibited expression patterns more closely aligned with those of DREB genes...” The gene expression data do not fully support this equivalence. Provide statistical support or rephrase more cautiously.

We sincerely thank the reviewer for this observation. We agree with your observation and have rephrased the sentence more cautiously.

“RD29A and RD29B are highly induced by various types of abiotic stress and are regulated primarily by ABA-dependent pathways. However, RD29A is also influenced by ABA-independent signaling mediated by DREB/CBF transcription factors. In our data, RD29A showed a partially overlapping expression trend with DREB genes, suggesting potential co-regulation, whereas RD29B exhibited a response more consistent with classical ABA-dependent signaling genes (Fig 9, J, K).”

6. Hygroscopic properties of fullerenol with no water retention data. Conduct thermogravimetric analysis or remove claim.

We acknowledge the reviewer’s concern regarding the lack of direct experimental evidence for water retention by fullerenol in the current study. While our current work does not include thermogravimetric analysis, our previously published study [66] demonstrated that FNPs can form self-assembled aggregates capable of interlocking water molecules, with temperature-dependent release from solvation layers. We now clarify in the discussion that

this represents a proposed mechanism that may contribute to improved drought resilience by providing a gradual water supply under low water potential conditions. This hypothesis, although mechanistically plausible, remains to be confirmed in future studies. We have modified the manuscript accordingly to frame this statement more cautiously, avoiding overinterpretation.

"These findings suggest that the combined antioxidant and the ability of FNPs to interact with water, attributable to their numerous surface hydroxyl (–OH) groups, might support increased photosynthetic rates, both when applied individually and with ZnO nanoparticles. The polarity of these hydroxyl groups facilitates hydrogen bonding with water molecules, potentially improving water availability and contributing to enhanced stomatal conductance under drought stress in both Col-0 and pp2ca-1 mutant plants."

7. Missing RIN values and amplification efficiency data (violates MIQE guidelines).

We thank the reviewer for their insightful observation. In our study, we initially assessed RNA quality and purity using a NanoDrop spectrophotometer by monitoring A260/280 and A260/230 ratios. Although we did not evaluate RNA integrity directly by evaluating RIN values, we introduced an additional quality control step by assessing the resulting cDNA with a Qubit fluorometer. Only samples meeting acceptable purity criteria (A260/280 > 1.7 and A260/230 > 2.2) were retained for qRT-PCR analysis. While these combined measures ensured the usability of our samples for downstream expression analysis, we acknowledge that a formal RNA integrity assessment would further strengthen our findings. Therefore, we will certainly include those analyses in our future research.

8. Manuscript lacks accessible raw data for reproducibility. Include supplementary tables with raw data.

We thank the reviewer for this suggestion; however, the raw data have been submitted as supplementary information (Table S1).

9. Statistical reporting is incomplete (missing F-values, degrees of freedom, p-values).

Thank you for noticing and pointing this out. It appears that during the submission process, the Supplementary Information file—which includes the datasets used and the outputs of

thorough statistical tests—was not properly linked to the submission. We have ensured that the file is included and accessible with the revised submission.

10. Use consistent terminology throughout: sometimes ZnO nano, other times ZnO NPs or ZnO nanoparticles.

We thank the reviewer for their keen observation. We have carefully reviewed and corrected the terminology throughout the manuscript to ensure consistency.

11. ENPs is undefined upon first use. Expand to engineered nanoparticles (ENPs) initially.

We are sorry for this lapse. We have defined the term according to the reviewer's valuable suggestion.

"Engineered nanoparticles (ENPs) are purpose-designed nanomaterials with at least one dimension below 100 nm, exhibiting tailored chemical and physical properties. Composed of metals, polymers, semiconductors, or carbon-based materials, ENPs are widely used in biomedicine, electronics, environmental and agricultural applications.
