# Supplementary material for: Foliar application of fullerenol and zinc oxide nanoparticles improves stress resilience in drought-sensitive Arabidopsis thaliana
Source: PLoS One. 2025 Aug 19;20(8):e0330022. doi: 10.1371/journal.pone.0330022 (PMC12364364; doi:10.1371/journal.pone.0330022)
Supplement: Attachment — Submitted filename: Review.docx [file pone.0330022.s002.docx]

**Abstract:**

Clarity and Focus:

- Comment: The abstract is generally well-written and informative, but it's slightly dense and could benefit from more concise phrasing to improve readability. Some sentences are overly long or complex.
- Suggestion: Break up long sentences to separate methodology from results, and group findings logically (e.g., physiological effects vs. molecular effects).

Terminology:

- Issue: Use of both “ZnO nanoparticles” and “ZnO nano” is inconsistent.
  - Recommendation: Use one term throughout, ideally “ZnO nanoparticles (ZnO NPs)” and define it early.

Structure and Flow:

- Comment: The paragraph is slightly long and would benefit from being split into two: one focusing on physiological findings, the other on molecular responses.

Minor suggestions:

- Change “drought stress acclimatization” → “drought acclimation” for conciseness.
- “Photosynthetic performance, stomatal conductance and water-use efficiency were optimized…” — consider rephrasing “optimized” to “enhanced” or “improved” for precision.

**Introduction**

Minor Language Edits

- “...generating resilient crops with increased yield and quality.” → “...yield and nutritional quality.”
- “stress-smart plants” → put in quotes only if directly quoted from a source.
- “It offers advantages over traditional soil fertilisation…” → “...fertilization,” if following US English.

**Materials & Methods**

Major Comments:

1. Clarity and Flow:
   - The section is comprehensive but densely packed. Consider breaking longer paragraphs (e.g., *"Nanoparticles characterization"*, *"Plant material and experimental design"*) into subsections or bulleted formats for easier readability.
   - Repeated phrases like “measured after prior sonication, simultaneously with foliar application” may benefit from rephrasing for smoother flow and clearer time sequencing.
2. **Justification for Methodology:**
   - Some methods lack a brief rationale. For example:
     - Why were certain concentrations of nanoparticles (e.g., 100 µmol/L fullerenol, 10 mg/L ZnO) selected? Were these based on previous studies or preliminary trials?
     - Why was the Photosynthesis RIDES 2.0 protocol used specifically? A short explanation would strengthen reproducibility and justification.
3. **Replication and Statistical Rigor:**
   - While replicates are mentioned (e.g., "12 replicates measured across four plants per treatment"), it would help to clarify whether these were biological or technical replicates in all cases.
   - Mentioning the randomization process or blocking (if done) in plant arrangement would improve transparency.
4. **Gene Expression:**
   - The description of ∆∆Ct is mostly clear, but it would benefit from stating if amplification efficiencies were verified and how they were normalized across genes.
   - Including references for the primer sequences or information on their validation (e.g., amplification efficiency or specificity via melt curves) would strengthen this section.
5. **Software & Statistical Interpretation:**
   - The mixed-effects model explanation is thorough and well-articulated, but readers unfamiliar with statistics may benefit from a flowchart or schematic in supplementary materials.
   - For PCA, it's excellent that Horn’s parallel analysis was used. A quick justification for retaining only the first two components, beyond the explained variance, would be useful.

**Minor Comments:**

- **Typos and Formatting:**
  - "encoding the Protein Phosphatase 2 C(PP2C)" → Add a space: “2 C (PP2C)”
  - "FNP100-ZnO nano" → Be consistent: use either “ZnO nano” or “nano ZnO” throughout.
  - "Photosynthetic rates (PN)" → Consider clarifying abbreviation earlier: PN = Net photosynthesis rate.
- **Consistency of Units and Style:**
  - Use consistent units: sometimes “µmol/L” and elsewhere “µmol L⁻¹” might appear in the full manuscript. Stick to one format.
  - Space between value and unit should be consistent: “10 mg/L” (correct), not “10mg/L”.
- **Referencing Style:**
  - Reference numbers [8,31] should ideally include a phrase like “as previously described by…” to contextualize them.
  - Ensure consistent citation of methods, especially those adapted or modified.

**Results & Discussion to Fig. 5. IR analysis of the FNP, ZnO nano, and FNP100-ZnO nano**

Minor Comments:

- Grammar & Style:
  - “ZnO nano particles” → should be “ZnO nanoparticles” consistently.
  - “ZnO nano and FNP are better visible” → consider “more clearly observed.”
  - “compelling electrostatic interactions” → consider “strong electrostatic interactions” for more formal tone.
- Terminology Consistency:
  - “nano ZnO”, “ZnO nano”, “ZnO nanoparticles” are used interchangeably. Choose one terminology and apply it consistently throughout the manuscript.
  - “molar ratio of FNP 100 μmol/ZnO nano 1.3 μmol” appears frequently; you may abbreviate it as “FNP100–ZnO1.3” after first mention for smoother reading.
- Units and Notation:
  - Use SI-compliant spacing: e.g., “100 μmol/L”, not “100μmol/L”.
  - Ensure all values (particle size, zeta potential, etc.) use consistent significant figures and unit formatting.

**Assessment of leaf phenotypic changes indicates NP specificities to Drought response gene expression is modulated in response to FNP and ZnO nano**

Minor Editorial Suggestions:

- Replace repetitive phrases like “interesting,” “remarkable,” or “notably” with more precise descriptors.
- Standardize treatment names (e.g., “FNP100-ZnO nano” vs. “ZnO nano + FNP100”) across the manuscript for consistency.
- Re-check abbreviations on first use (e.g., SPAD, PN, gs) to ensure clarity for readers outside the immediate field.

**Up to conclusion:**

**Minor Typo:**

- In the sentence "On the contrary, In the pp2ca-1 mutant," the "In" should not be capitalized.

**Conclusion**

The conclusion does a good job of summarizing the findings and highlighting their importance, particularly for practical applications in agriculture. It could be strengthened with more explicit links to the study's objectives and clearer explanations of the observed synergistic effects.
