# Supplementary material for: Foliar application of fullerenol and zinc oxide nanoparticles improves stress resilience in drought-sensitive Arabidopsis thaliana
Source: PLoS One. 2025 Aug 19;20(8):e0330022. doi: 10.1371/journal.pone.0330022 (PMC12364364; doi:10.1371/journal.pone.0330022)
Supplement: Attachment — Submitted filename: Comments.docx [file pone.0330022.s003.docx]

Comments

The manuscript “Foliar application of fullerenol and zinc oxide nanoparticles improves stress resilience in drought-sensitive Arabidopsis thaliana” presents a detailed and thorough study about characterization and chemical properties of fullerenol nanoparticles (FNP) and zinc oxide nanoparticles (ZnO nano). Arabidopsis thaliana is an interesting choice to undertake these studies as it is regarded as model plant to study biology, stress response and genetics. As a result of nanoparticle treatment, study about changes in gene expression emphasized synergistic impact of combining ZnO nanoparticles with FNPs. However, a few queries need to be addressed to make this manuscript more rigorous.

1. Hydroxylated fullerenes have already been studied extensively for foliar applications previously. What leads to choice of fullerenol in your research?
2. In introduction, are ENPs, engineered nanoparticles? Explain a little about ENPs to make the readers better understand them.
3. What is the rationale behind choosing fullerenes with 24 hydroxyl groups? As 18 to 40 hydroxyl groups is considered the practical range for most applications, why did the study not consider fullerenols with different numbers of functional groups?
4. Under the heading of Chemicals Used, synthesis of fullerenol NPs has not been explained. It is not appropriate to present it as a reference.
5. In topic, RNA Isolation and quantitative RT-PCR analysis, how the RNA quality and purity ensured?
6. The results of SEM, DLS, Zeta potential, XRD Diffractograms, and FTIR of the FNP100-ZnO nano combination effectively characterize their formation.
7. During experiment were TBARS levels correlated statistically with non-enzymatic antioxidants like proline/glutathione?
8. Effects of different ZnO nanoparticle concentrations on plant growth and stress resistance are reviewed extensively, quoting numerous references. It looks like little overemphasized in comparison to FNP100-ZnO nano combination.
9. What is the rationale behind excluding any other plant hormone pathways, like auxin or salicylic acid for studying the effects of nanoparticles on plant stress responses?
